# Burden of colorectal cancer attributable to dietary risks in China from 1990 to 2021: findings from the Global Burden of Disease Study 2021

**DOI:** 10.3389/fnut.2025.1673267

**Published:** 2026-01-06

**Authors:** Bijuan Chen, Hanchen Zheng, Rui Huang, Jiami Yu, Xiaojie Wang, Chunkang Yang, Zengqing Guo, Zhouwei Zhan

**Affiliations:** 1 Department of Radiation Oncology, Clinical Oncology School of Fujian Medical University, Fujian Cancer Hospital, Fuzhou, Fujian, China; 2 Department of Medical Oncology, Clinical Oncology School of Fujian Medical University, Fujian Cancer Hospital, Fuzhou, Fujian, China; 3 Digestive Endoscopy Center, Clinical Oncology School of Fujian Medical University, Fujian Cancer Hospital, Fuzhou, Fujian, China; 4 Department of Gastrointestinal Surgery, Clinical Oncology School of Fujian Medical University, Fujian Cancer Hospital, Fuzhou, Fujian, China

**Keywords:** colorectal cancer, dietary risk, China, Global Burden of Disease, age-period-cohort analysis, decomposition analysis

## Abstract

**Background:**

Colon and rectum cancer (CRC) poses a major public health challenge in China, with dietary risks identified as key modifiable contributors to its incidence and mortality. Quantifying the current burden, long-term trends, and the impact of specific dietary factors is essential to inform effective prevention strategies.

**Methods:**

Data on CRC burden attributable to dietary risks in China from 1990 to 2021 were extracted from the Global Burden of Disease Study 2021. We examined all-age numbers and age-standardized rates of CRC deaths, disability-adjusted life years (DALYs), years lived with disability (YLDs), and years of life lost (YLLs). Temporal trends and generational patterns were explored using joinpoint regression, age-period-cohort, and decomposition analyses.

**Results:**

In 2021, dietary risks accounted for over 102,000 CRC-related deaths and 2.55 million DALYs in China, with a significantly higher burden among males. Although age-standardized mortality, DALY, and YLL rates declined modestly over the past three decades, YLD rates increased. Diets low in milk, whole grains, and calcium were the predominant contributors, while the burden associated with processed meat showed the largest relative increase. The highest burden occurred among those aged 65–74, with notable sex disparities. Joinpoint analysis revealed fluctuating trends, including recent plateaus or increases, particularly in males. Age-period-cohort analysis indicated elevated burden in earlier birth cohorts, with improvements in more recent generations. Decomposition analysis identified population aging as the primary driver of burden growth, whereas epidemiological changes contributed modest reductions, especially among women.

**Conclusion:**

The burden of CRC attributable to dietary risks remains considerable in China, underscoring the need for targeted, gender- and age-sensitive dietary interventions to reduce premature mortality and long-term disability.

## Introduction

Colon and rectum cancer (CRC) is one of the most commonly diagnosed malignancies and a leading cause of cancer-related deaths worldwide (1, 2). In recent decades, its incidence has shifted markedly from high-income nations to low- and middle-income countries, including China, where demographic transitions, lifestyle changes, and westernized diets have contributed to rising disease burdens (3–6). According to estimates from the Global Burden of Disease (GBD) Study, CRC remains among the top contributors to cancer mortality in China, with a substantial proportion of its burden attributable to modifiable risk factors, particularly dietary risks (5). Despite stable or declining age-standardized CRC rates in some regions, absolute deaths and disability-adjusted life years (DALYs) continue to rise in China due to population aging and poor diets (5, 7). Clarifying the role of specific dietary factors is key to designing tailored prevention strategies.

Suboptimal dietary intake has been identified as a key modifiable risk factor for CRC (8). Specifically, diets low in fiber, calcium, milk, and whole grains, as well as those high in red or processed meat, have consistently been associated with increased CRC risk (9–12). These associations are supported by biological mechanisms involving gut microbiota dysbiosis, chronic inflammation, and carcinogenic compound formation during meat processing (13–15). In China, rapid urbanization and dietary westernization have led to a nutritional transition characterized by a shift away from traditional diets rich in coarse grains and legumes toward increased consumption of refined grains, edible oils, sugar-sweetened beverages, fried foods, and animal-source products such as pork, processed meats, and eggs (16, 17). These changes reflect a broader movement toward energy-dense, low-fiber diets with reduced diversity in plant-based foods. Recent population-based studies show that these dietary shifts have had a measurable impact on CRC-related outcomes, particularly among older adults and male populations who typically consume more red and processed meats and fewer protective dietary components such as whole grains, fiber, and dairy (17). Despite growing recognition of the role of diet in CRC, limited research has quantified the evolving impact of dietary risks on CRC burden over time in China.

This study aims to evaluate the long-term trends and demographic patterns of CRC burden attributable to dietary risks in China from 1990 to 2021. Drawing on nationally representative data, we focus on the evolution of mortality and disability associated with diet-related CRC across different age and sex groups. The study also explores how individual dietary components have contributed to the burden over time and situates national findings within a global context. By clarifying these patterns, our objective is to inform targeted dietary and public health strategies that can reduce preventable morbidity and mortality, particularly in light of China’s aging population and shifting nutritional landscape.

## Methods

### Data source

Data were obtained from the GBD 2021, a comprehensive initiative led by the Institute for Health Metrics and Evaluation (IHME) to quantify health loss from diseases, injuries, and risk factors across 204 countries and territories between 1990 and 2021 (18, 19). For China, GBD estimates were derived from a range of data sources, including original epidemiologic studies, national surveys (e.g., the China Disease Surveillance Points system), cancer registries, hospital records, and published literature. However, the representativeness of these data may vary across regions, as cancer registry coverage in China tends to be more comprehensive in urban areas than in rural settings. This imbalance may lead to potential underrepresentation of rural populations and contribute to regional estimation bias. These data inputs were synthesized using standardized GBD modeling tools to ensure consistency and comparability. Specifically, data on CRC attributable to dietary risks in China were extracted, including the number and age-standardized rates (ASRs) of deaths, DALYs, years lived with disability (YLDs), and years of life lost (YLLs). Six dietary risk components were evaluated: diets high in red meat or processed meat, and diets low in milk, calcium, fiber, and whole grains, based on GBD-defined risk-exposure-outcome pairs (18). Estimates were generated using DisMod-MR 2.1, a Bayesian meta-regression tool that ensures internal consistency and accounts for data heterogeneity (18, 20). The comparative risk assessment (CRA) framework was used to quantify the risk-attributable burden by estimating the proportion of disease that could be avoided if exposure were reduced to the theoretical minimum risk exposure level (TMREL). Publicly available data were accessed through the Global Health Data Exchange (GHDx) platform.

### Definition and estimation

CRC was defined by ICD-10 codes C18-C21. Dietary risk burden was estimated using the CRA framework by comparing current exposures to TMRELs, the optimal intake linked to minimal disease risk (18, 20). The intake ranges and TMRELs for each food group were defined by the GBD Study 2021 and reflect global evidence-based thresholds (18). The six dietary risk factors were operationalized as follows: A diet low in whole grains was defined as <160–210 g/day of intact grains (bran, germ, endosperm) from sources such as cereals, bread, and rice. Low milk intake was <500–610 g/day for females and <280–340 g/day for males, excluding plant-based alternatives. A high red meat diet involved >0 g/day (95% UI: 0–200) of unprocessed beef, pork, lamb, or goat. A low fiber diet was <22–25 g/day from fruits, vegetables, legumes, and grains. Low calcium intake was <1.1–1.2 g/day for females and <0.72–0.86 g/day for males. A high processed meat diet included any intake of meats preserved by smoking, curing, salting, or chemical preservatives (e.g., sausages, bacon, cold cuts). Population-attributable fractions (PAFs) were calculated using relative risks from meta-analyses, exposure distributions, and TMRELs. These PAFs were applied to cause-specific CRC burden to estimate deaths, DALYs, YLDs, and YLLs attributable to each dietary risk. Modeling was conducted using DisMod-MR 2.1 to ensure consistency and account for uncertainty.

### Descriptive analysis

Temporal trends in CRC burden attributable to dietary risks in China from 1990 to 2021 were assessed descriptively. Burden was quantified by the number and ASRs of deaths, DALYs, YLDs, and YLLs per 100,000 population. Data were retrieved from the GBD 2021 database via the GHDx platform, and age-standardization was performed using the GBD reference population (18, 20). ASRs (per 100,000 people) were calculated using the following formula: $\mathrm{ASR}=\frac{\sum_{i=1}^{N}\mathrm{ai}*\mathrm{\omega i}}{\sum_{i=1}^{N}\mathrm{\omega i}}\times 100,000$ , where *ai* represents the age-specific rate in the *i* th age group, and *ωi* is the number (or weight) of individuals in the corresponding age group of the standard population. The GBD world standard population was used for weighting, consistent with GBD 2021 methodology (21). Data were stratified by sex, age group, year, and dietary risk factor. Estimates for 2021 were compared with 1990 to evaluate changes over time. Age-specific distributions were analyzed for both years to assess shifts in burden across life stages. Leading dietary contributors were identified by stratifying data by risk factor. Crude and ASRs were presented with 95% uncertainty intervals (UIs), representing the 2.5th and 97.5th percentiles of 1,000 posterior draws. Trends were visualized using line and bar graphs to illustrate time-series patterns, sex differences, and age variations. These descriptive findings provided a foundation for further analytical modeling.

### Joinpoint regression analysis

To quantify temporal trends in the burden of CRC attributable to dietary risks in China from 1990 to 2021, Joinpoint regression analysis was applied to ASRs of deaths, DALYs, YLDs, and YLLs. This method detects statistically significant inflection points in temporal trends, known as joinpoints, enabling the calculation of the annual percentage change (APC) for each distinct time segment, as well as the average annual percentage change (AAPC) across the entire study period (22, 23). The analysis was conducted using Joinpoint Regression Program version 5.2.0, developed by the US National Cancer Institute. A maximum of five joinpoints were allowed for each regression model. The Monte Carlo Permutation method was used to determine the number of joinpoints, with statistical significance set at *p* < 0.05. APCs and AAPCs were reported with corresponding 95% confidence intervals (CIs) to assess whether trends were increasing, decreasing, or stable. Separate models were fitted for each outcome (deaths, DALYs, YLDs, and YLLs) and stratified by sex. This allowed us to evaluate sex-specific temporal patterns and to determine whether disease burden trends differed across genders. The analysis provided key insights into time periods when substantial shifts in the burden occurred, and whether such shifts aligned with national public health efforts or changes in population dietary behavior.

### Age-period-cohort (APC) analysis

To disentangle the independent effects of age, time period, and birth cohort on CRC mortality and DALYs attributable to dietary risks in China, an APC analysis was performed. This approach is essential to evaluate temporal trends influenced by demographic aging, changing exposure patterns, and generational shifts in dietary behavior and healthcare access. Given the collinearity among age, period, and cohort, we applied the intrinsic estimator (IE) method, which provides statistically consistent and interpretable parameter estimates in APC modeling frameworks (5, 24). For the mortality analysis, the population was divided into 5-year age groups ranging from 15–19 to 95+, while for the DALY analysis, age was grouped from 25–29 to 95+. Calendar time was stratified into consecutive five-year periods (1992–1996 through 2017–2021), and birth cohorts were derived accordingly. The youngest and oldest age categories were grouped at the extremes to ensure model stability and comparability. The APC analysis was conducted using the “Epi” package (version 2.46) in R software (version 4.3.1). Model diagnostics included the evaluation of residual deviance and Akaike Information Criterion (AIC) to determine optimal fit. These analyses enabled a clearer understanding of how age, temporal shifts, and generational exposures contribute to the evolving burden of CRC linked to dietary factors.

### Decomposition analysis

To quantify the relative contributions of population growth, population aging, and changes in epidemiological factors to the net change in CRC burden attributable to dietary risks in China from 1990 to 2021, we performed a decomposition analysis. This method provides a structured approach to isolate how demographic and non-demographic factors influence the trends in mortality and DALYs, offering insights into the underlying drivers of observed changes over time. The decomposition analysis was based on the Das Gupta method, which has been widely used in global burden studies to disentangle the effects of population size, age structure, and age-specific disease rates on overall disease burden changes (22). In this study, the total change in CRC deaths and DALYs between 1990 and 2021 was separated into three components: (1) population growth (reflecting an increase in the total number of individuals), (2) population aging (reflecting the shift toward older age groups), and (3) changes in age-specific and cause-specific mortality and DALY rates (representing epidemiological transitions, including exposure reduction, improved screening, and treatment advances). Sex-specific analyses were conducted to reveal gender-related differences in the contribution of each factor. Results were presented in absolute numbers and proportions to demonstrate the magnitude of each component’s effect.

## Results

### Current burden of CRC attributable to dietary risks in China, 2021

In 2021, dietary risks continued to substantially contribute to the CRC burden in China. An estimated 102,694 CRC deaths and over 2.55 million DALYs were attributable to suboptimal dietary patterns, with men bearing a disproportionately higher burden than women. Age-standardized mortality and DALY rates were significantly higher among males (6.78 and 161.43 per 100,000, respectively) compared to females (3.72 and 87.55 per 100,000). This gender gap may reflect differences in dietary habits, health behaviors, or access to healthcare. The vast majority of DALYs were driven by YLLs, underscoring the high rate of premature mortality associated with diet-related CRC. Although YLDs contributed less to total DALYs, they still reflected the ongoing impact of CRC-related chronic disability, particularly among older adults. Overall, the findings emphasize the persistent and gender-skewed health burden from diet-related CRC in China, highlighting the need for targeted public health strategies (Table 1).

**Table 1:** All-age cases and age-standardized deaths, DALYs, YLDs, and YLLs rates in 2021 for CRC attributable to dietary risks in China.

Measure	All-ages cases (95% UI)			Age-standardized rates per 100,000 people (95% UI)		
	Total	Male	Female	Total	Male	Female
Deaths	102,694 (36,137, 166,537)	62,503 (19,457, 104,206)	40,191 (16,370, 64,866)	5.08 (1.79, 8.21)	6.78 (2.17, 11.32)	3.72 (1.51, 5.99)
DALYs	2,555,445 (880,337, 4,134,442)	1,609,657 (488,436, 2,656,291)	945,787 (380,533, 1,539,248)	122.93 (42.56, 198.49)	161.43 (49.60, 266.77)	87.55 (35.22, 142.57)
YLDs	122,902 (40,020, 214,432)	75,740 (21,673, 136,737)	47,162 (17,383, 82,627)	5.79 (1.89, 10.07)	7.41 (2.13, 13.30)	4.29 (1.58, 7.54)
YLLs	2,432,542 (842,811, 3,948,619)	1,533,917 (468,338, 2,556,456)	898,625 (362,457, 1,454,720)	117.14 (40.78, 189.89)	154.02 (47.35, 257.01)	83.26 (33.57, 134.79)

Values in parentheses indicate 95% UIs, estimated using Monte Carlo simulations. CRC, colon and rectum cancer; DALYs, disability-adjusted life-years; YLDs, years lived with disability; YLLs, years of life lost; UI, uncertainty interval. GBD uncertainty precludes valid male–female hypothesis testing; no p values reported.

### Age and sex patterns of CRC burden attributable to dietary risks in China, 2021

**Figure 1:**
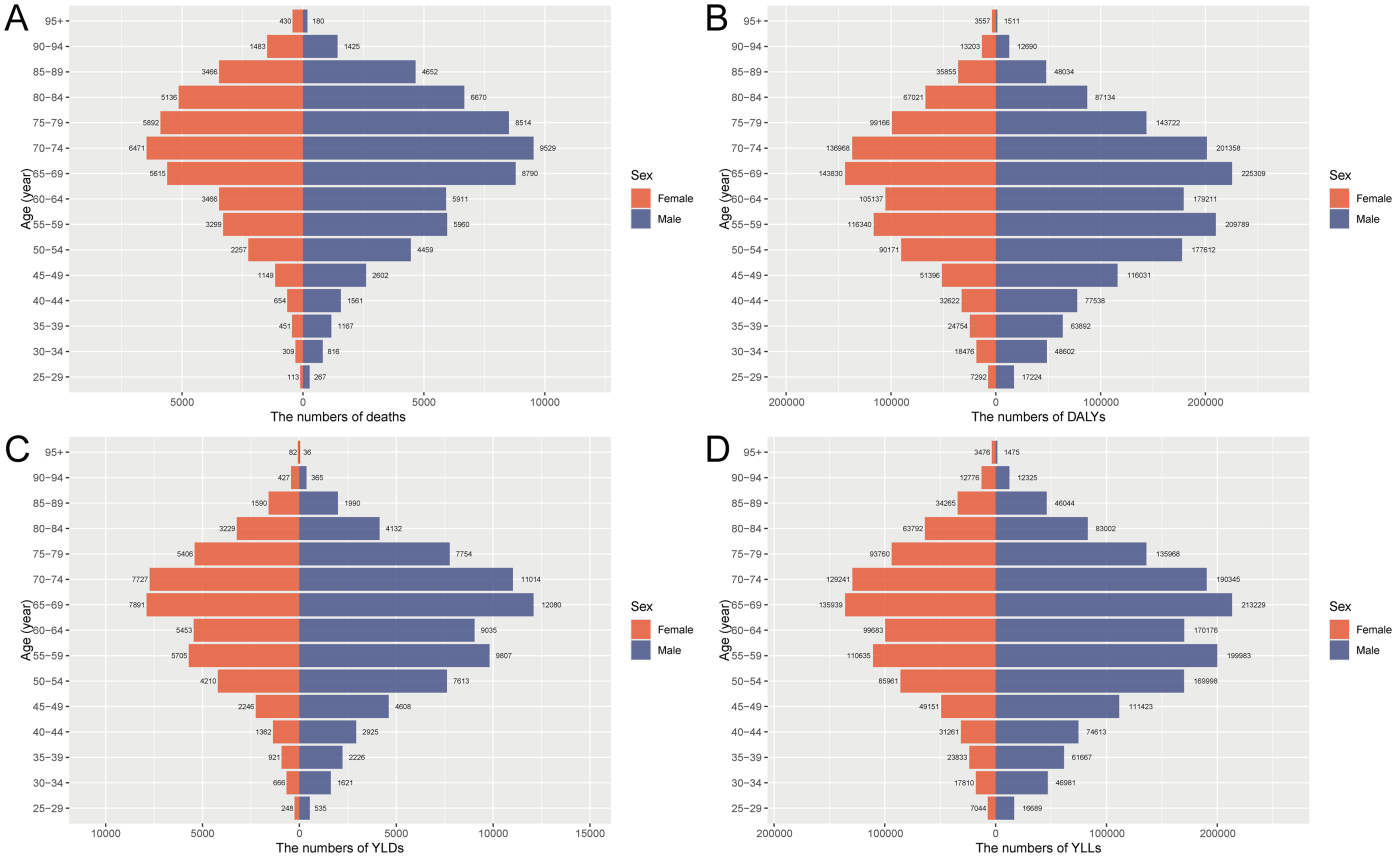
Age- and sex-specific numbers of **(A)** deaths, **(B)** DALYs, **(C)** YLDs, and **(D)** YLLs for CRC attributable to dietary risks in China, 2021. DALYs, disability-adjusted life years; YLDs, years lived with disability; YLLs, years of life lost; CRC, colon and rectum cancer.

**Figure 2:**
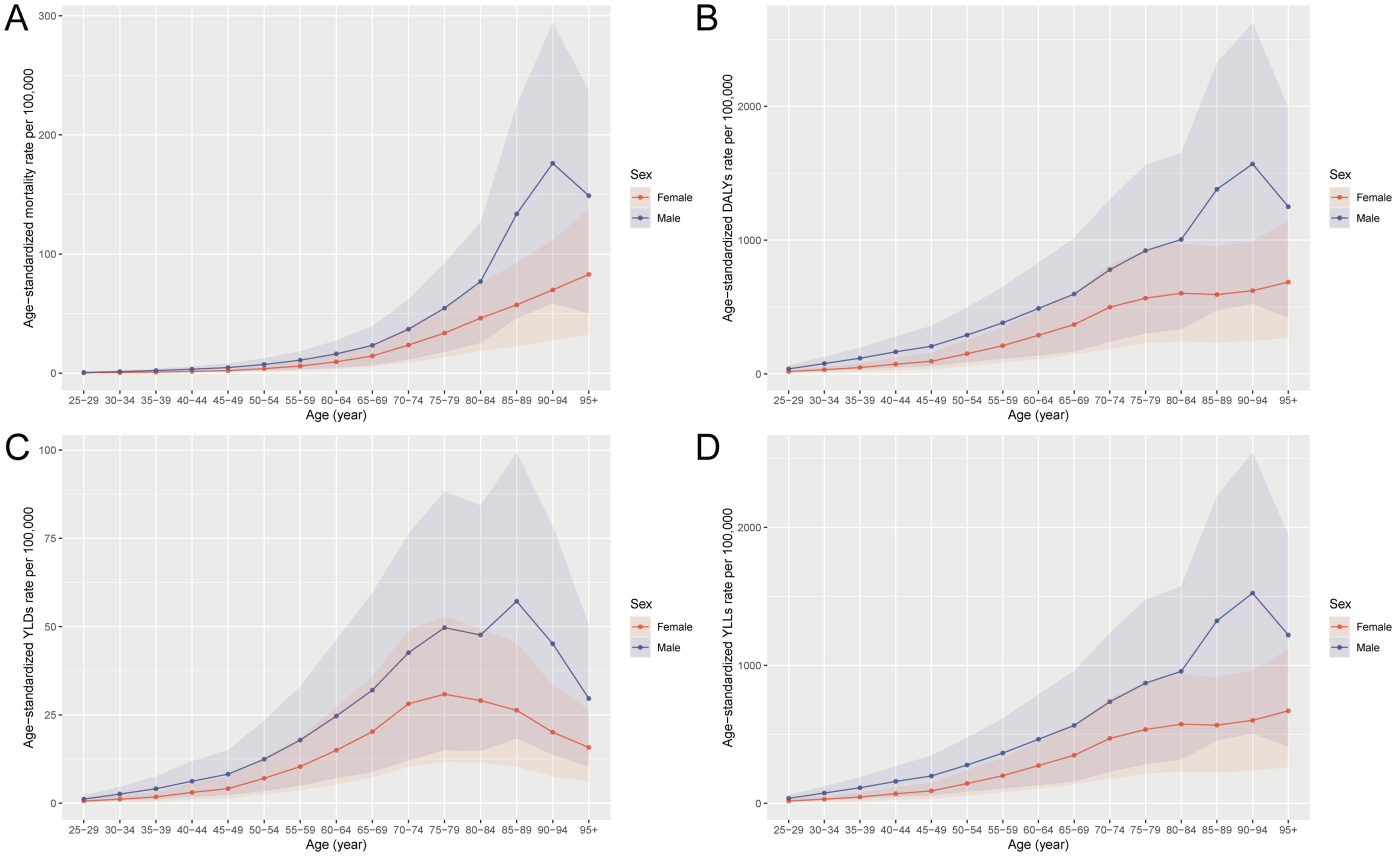
Age-specific and sex-specific age-standardized rates of **(A)** mortality, **(B)** DALYs, **(C)** YLDs, and **(D)** YLLs for CRC attributable to dietary risks in China, 2021. DALYs, disability-adjusted life years; YLDs, years lived with disability; YLLs, years of life lost.

In 2021, the burden of CRC attributable to dietary risks in China showed pronounced disparities by age and sex. The overall burden increased markedly with advancing age, peaking among individuals aged 65–74 years. Males consistently exhibited higher levels of deaths, YLLs, YLDs, and DALYs than females, underscoring a greater vulnerability likely linked to behavioral and biological factors. Mortality and YLL rates rose sharply after age 50 and were highest in the 85–94 age group, with men showing significantly steeper increases. DALY patterns largely mirrored those of mortality, reflecting the dominance of premature death in CRC burden. Although YLDs accounted for a smaller share, they still represented a meaningful and persistent impact of CRC-related disability, especially in older age groups. These sex-based differences were evident across all burden indicators and emphasize the importance of designing dietary interventions tailored to high-risk male populations. Overall, the data highlight aging and male sex as critical risk modifiers in dietary-related CRC burden, warranting targeted preventive efforts (Figures 1, 2).

### Temporal trends in CRC burden attributable to dietary risks in China, 1990–2021

**Figure 3:**
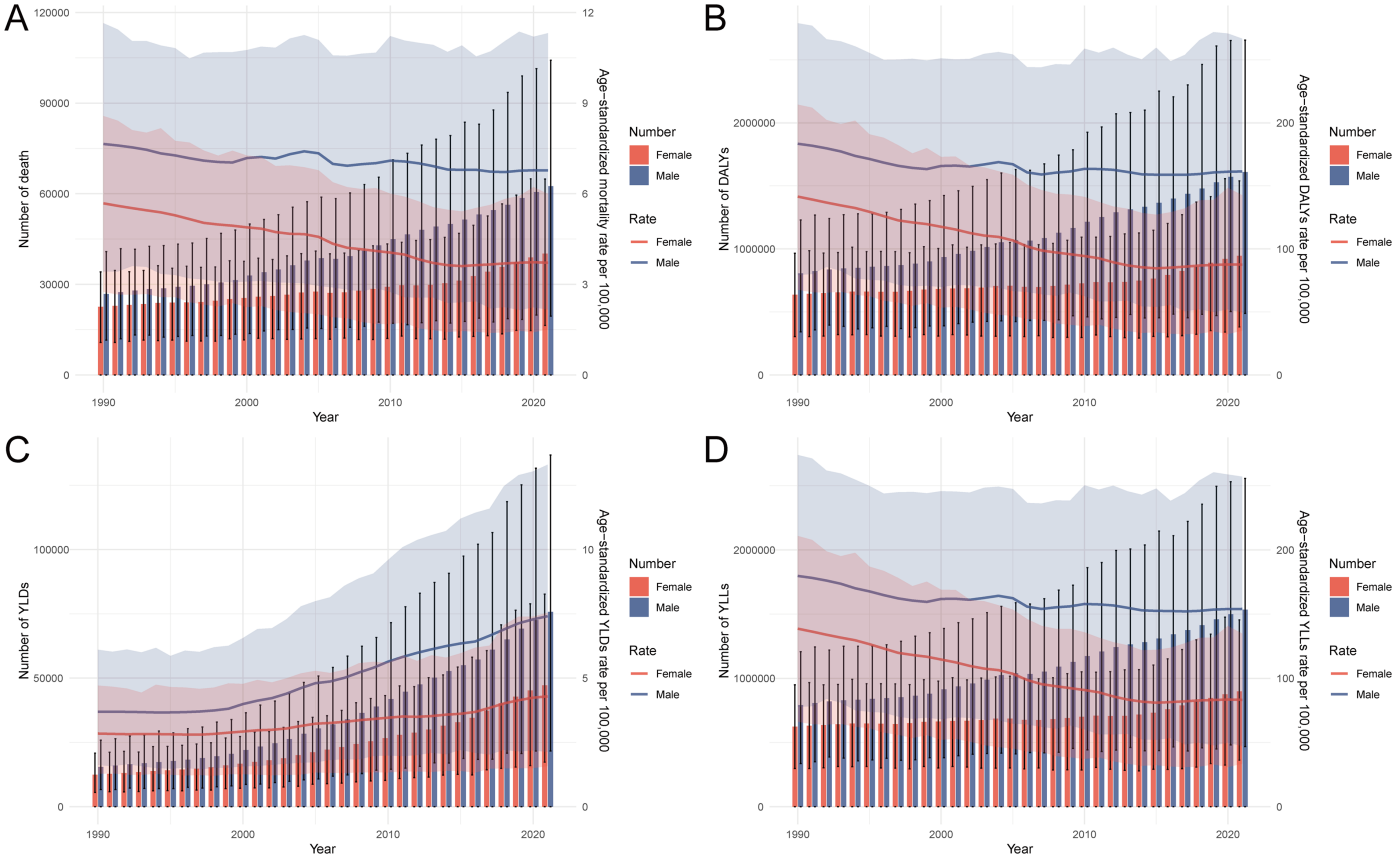
Trends in numbers and age-standardized rates of **(A)** deaths, **(B)** DALYs, **(C)** YLDs, and **(D)** YLLs for CRC attributable to dietary risks in China, 1990–2021, by sex. DALYs, disability-adjusted life years; YLDs, years lived with disability; YLLs, years of life lost; CRC, colon and rectum cancer.

From 1990 to 2021, the burden of CRC attributable to dietary risks in China increased markedly in absolute terms, with persistent disparities across sex and age groups. Figure 3 illustrates a steady rise in the number of deaths, DALYs, YLDs, and YLLs in both sexes, particularly among males. Despite this growth, age-standardized rates for mortality, DALYs, and YLLs remained relatively stable or showed modest declines, suggesting that the overall rise in burden was largely driven by demographic transitions, such as population aging and expansion, rather than increased exposure to dietary risk factors. In contrast, YLDs exhibited a gradual increase both in absolute number and rate, reflecting the growing contribution of long-term disability associated with CRC. Supplementary Figure S1 supports this trend, showing higher numbers of CRC-related deaths, DALYs, and YLLs across most age groups in 2021, especially among those aged 45 years and older. Although crude rates of mortality, DALYs, and YLLs in 2021 were generally lower than in 1990, the crude rates of YLDs in most age groups were elevated, indicating a rising burden of chronic disability linked to dietary risk factors (Figure 3 and Supplementary Figure S1).

### Contribution of specific dietary risks to CRC burden in China, 1990–2021

**Figure 4:**
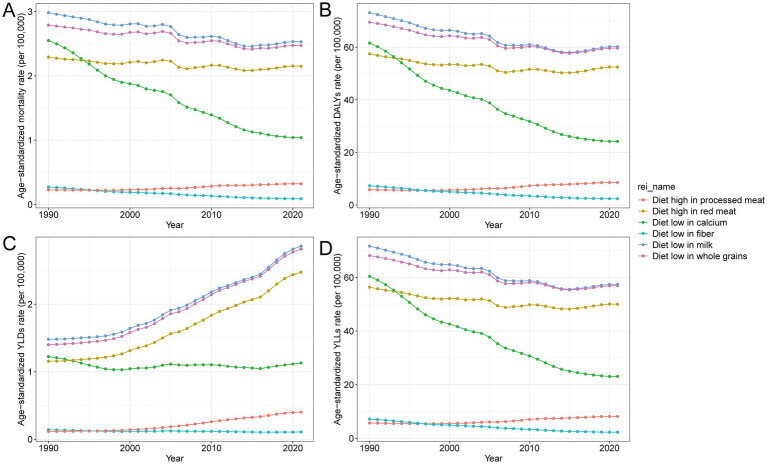
Trends in age-standardized rates of **(A)** mortality, **(B)** DALYs, **(C)** YLDs, and **(D)** YLLs for CRC attributable to different dietary risks in China, 1990–2021. The figure presents temporal changes in age-standardized rates per 100,000 population associated with six major dietary risks: diet high in processed meat, diet high in red meat, diet low in milk, diet low in calcium, diet low in fiber, and diet low in whole grains. DALYs, disability-adjusted life years; YLDs, years lived with disability; YLLs, years of life lost; CRC, colon and rectum cancer.

In 2021, the burden of CRC in China attributable to dietary risks was primarily linked to inadequate consumption of milk, whole grains, and calcium. These dietary components consistently ranked among the top contributors across the study period, reflecting their central role in CRC prevention. While notable progress has been made in reducing the burden associated with low fiber and calcium intake, particularly in terms of premature mortality, other concerning trends have emerged. Diets high in processed meat have shown the most significant relative increase in attributable burden since 1990, likely reflecting shifts in dietary behavior influenced by rapid urbanization and changing food environments. Although the contribution of low calcium intake has decreased, the sustained impact of low milk and whole grain consumption continues to pose a major public health concern. The coexistence of persistent deficiencies in protective nutrients and increasing exposure to harmful dietary factors points to a complex nutritional landscape that requires coordinated public health interventions focused on both reducing risks and promoting protective dietary habits (Table 2; Figure 4; Supplementary Table S1).

**Table 2:** Mortality and DALYs for CRC attributable to dietary risks in China, 2021, with trends in ASRs per 100,000 population, 1990–2021.

Dietary risk	Deaths			DALYs		
	No, in thousands	Age-standardized rate per 100,000	Percentage change from 1990 to 2021	No, in thousands	Age-standardized rate per 100,000	Percentage change from 1990 to 2021
Diet high in processed meat	6.6 (−1.4, 14.8)	0.3 (−0.1, 0.7)	41.7 (9.1, 85.8)	178.2 (−37, 405)	8.6 (−1.8, 19.3)	47 (12.5, 94.8)
Diet high in red meat	43.6 (0, 92.1)	2.1 (0, 4.5)	−6.2 (−27.4, 664.8)	1091.8 (−0.5, 2295.8)	52.5 (0, 110.3)	−8.7 (−30.7, 688.3)
Diet low in milk	51 (13.9, 86.8)	2.5 (0.7, 4.3)	−15.2 (−34, 7.7)	1253.6 (337.5, 2128.1)	60.3 (16.2, 102.2)	−17.6 (−36.9, 7.3)
Diet low in calcium	20.7 (14.6, 28.3)	1 (0.7, 1.4)	−59.3 (−68.8, −47.5)	500.5 (356.2, 682.9)	24.2 (17.1, 33.1)	−60.7 (−70.4, −47.8)
Diet low in fiber	1.7 (0.7, 3)	0.1 (0, 0.2)	−67.3 (−78.2, −54.6)	48.1 (18.9, 84.8)	2.4 (0.9, 4.2)	−67.2 (−78.6, −52.6)
Diet low in whole grains	50 (20.1, 79.9)	2.5 (1.0 3.9)	−11.5 (−32.4, 12.4)	1241.9 (503.2, 1978.5)	59.7 (24.2, 94.9)	−14.1 (−35.5, 10.5)

Values in parentheses indicate 95% UIs estimated from Monte Carlo simulations using GBD posterior draws. ASRs are per 100,000 population and age-standardized to the GBD standard population. CRC, colon and rectum cancer; DALYs, disability-adjusted life years; ASRs, age-standardized rates; UI, uncertainty interval.

### Comparison of CRC burden trends in China and globally, 1990–2021

Between 1990 and 2021, the age-standardized burden of CRC attributable to dietary risks in China demonstrated an overall declining trend, aligning with global progress in CRC prevention and control. Notable improvements were observed in mortality and DALY indicators (*p* < 0.001), largely driven by reductions in premature deaths, suggesting enhanced effectiveness of early detection and treatment. However, the decline in China was less pronounced than global averages, pointing to potential gaps in dietary risk mitigation and cancer care infrastructure. Unlike other indicators, age-standardized YLD rates increased in China (*p* < 0.001), more sharply than global trends, reflecting the growing number of CRC survivors living with long-term disability. This pattern indicates a shifting burden from fatal to chronic outcomes, underscoring the rising importance of survivorship care. These findings highlight the need to balance mortality reduction efforts with policies addressing rehabilitation, quality of life, and dietary interventions for CRC survivors to meet the demands of a transitioning disease burden (Table 3 and Supplementary Figure S2).

**Table 3:** Change of age-standardized rates in deaths, DALYs, YLDs, and YLLs for CRC attributable to dietary risks between 1990 and 2021 in China and global level.

Measure	China				Global			
	1990	2021	AAPC	*p-*value	1990	2021	AAPC	*p-*value
Deaths	6.5 (3.09, 9.63)	5.08 (1.79, 8.21)	−0.81 (−0.84 – −0.78)	0.000 ^***^	6.33 (2.26, 9.47)	4.82 (1.64, 7.46)	−0.88 (−0.90 – −0.86)	0.000 ^***^
DALYs	160.95 (74.99, 237.30)	122.93 (42.56, 198.49)	−0.88 (−0.91 – −0.85)	0.000 ^***^	144.88 (53.10, 215.52)	109.71 (37.68, 168.52)	−0.87 (−0.89 – −0.84)	0.000 ^***^
YLDs	3.23 (1.44, 5.22)	5.79 (1.89, 10.07)	1.92 (1.87–1.96)	0.000 ^***^	4.42 (1.32, 7.43)	4.87 (1.38, 8.17)	0.33 (0.32–0.35)	0.000 ^***^
YLLs	157.72 (73.42, 233.30)	117.14 (40.78, 189.89)	−0.97 (−1.00 –−0.94)	0.000 ^***^	140.46 (51.70, 209.70)	104.83 (36.20, 161.66)	−0.93 (−0.95 – −0.91)	0.000 ^***^

Values in parentheses indicate 95% UIs, estimated using Monte Carlo simulations. Abbreviations: CRC, colon and rectum cancer; DALYs, disability-adjusted life-years; YLDs, years lived with disability; YLLs, years of life lost; AAPC, average annual percent change presented for full period; AAPC and p from joinpoint regression (permutation test). Significance codes: *p* < 0.001 (***). Symbols shown for APC within periods and AAPC overall; exact *p*-values available upon request.

### Joinpoint trend analysis of CRC burden attributable to dietary risks in China

**Figure 5:**
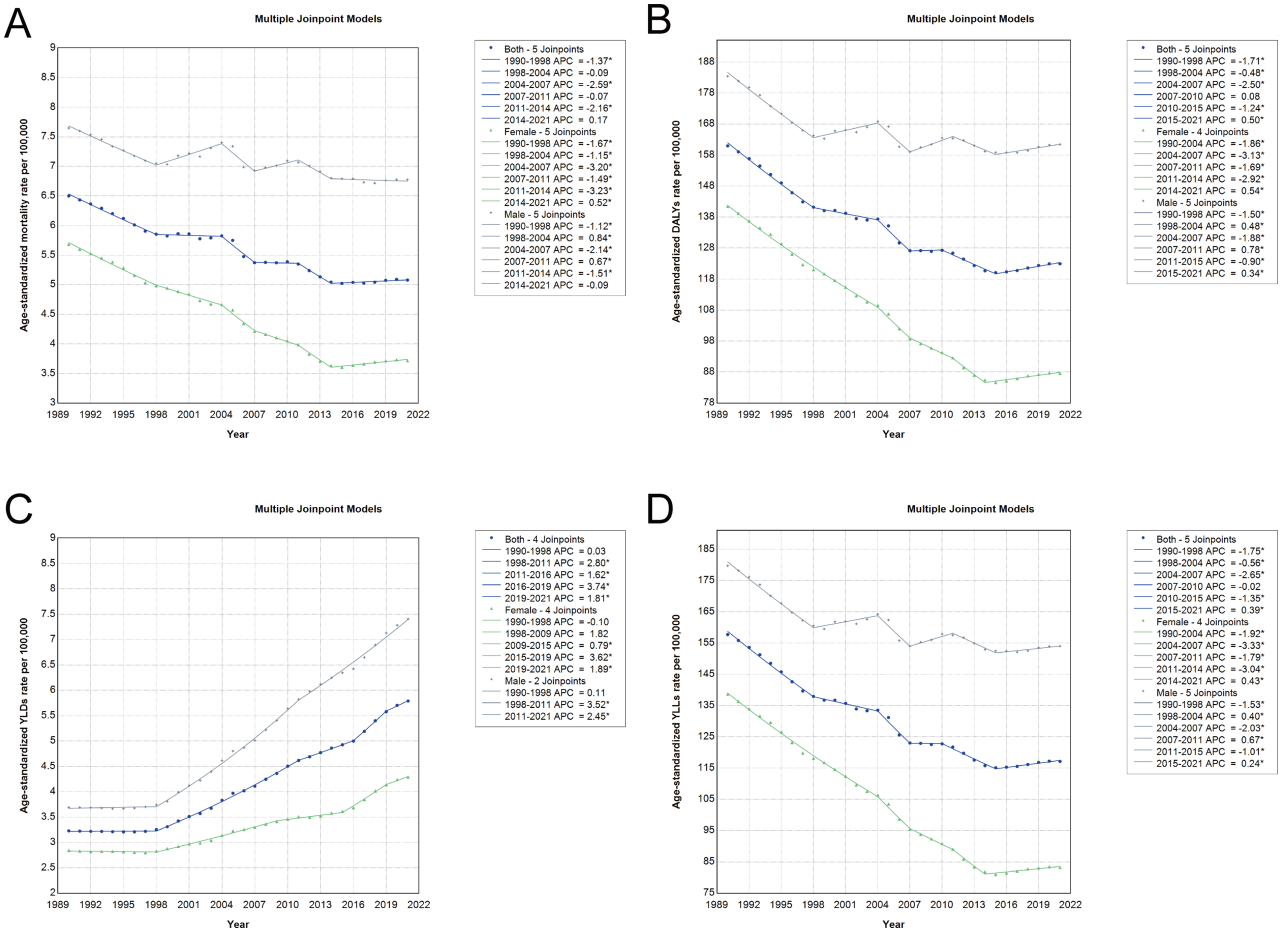
Joinpoint regression analysis of age-standardized rates for CRC attributable to dietary risks in China, 1990–2021. **(A)** Age-standardized mortality rate, **(B)** age-standardized DALY rate, **(C)** age-standardized YLD rate, and **(D)** age-standardized YLL rate per 100,000 population. Each panel illustrates joinpoint regression trajectories for the total population (blue line), females (green line), and males (gray line), with solid lines depicting modeled trends. Joinpoints mark statistically significant shifts in the direction or rate of change across the study period. APC, annual percentage change; DALY, disability-adjusted life year; YLD, years lived with disability; YLL, years of life lost; CRC, colon and rectum cancer.

Joinpoint regression analysis revealed several pivotal shifts in the age-standardized burden of CRC attributable to dietary risks in China from 1990 to 2021. A marked decline in mortality, DALY, and YLL rates was observed between 1990 and 1998 (*p* < 0.001), indicating early progress in public health measures. Another period of decline occurred from 2004 to 2007 (*p* < 0.001), suggesting potential reinforcement of interventions or health system improvements during that interval. However, these gains were not sustained uniformly, as rates plateaued or slightly increased after 2014, particularly in mortality and YLLs, signaling a stagnation in reducing premature deaths. In contrast, the burden of YLDs steadily rose throughout the study period (*p* < 0.001), reflecting a growing challenge in managing chronic sequelae of CRC. Among males, reductions in mortality and YLLs were less consistent, and the period from 1998 to 2004 showed an uptick rather than improvement. Notably, the YLD burden in men rose significantly after 1998 (*p* < 0.001), with a sharper increase from 1998 to 2011 compared to women, who experienced more gradual changes (Figure 5 and Supplementary Table S2).

### Age-period-cohort (APC) effects on mortality and DALYs for CRC attributable to dietary risks in China

**Figure 6:**
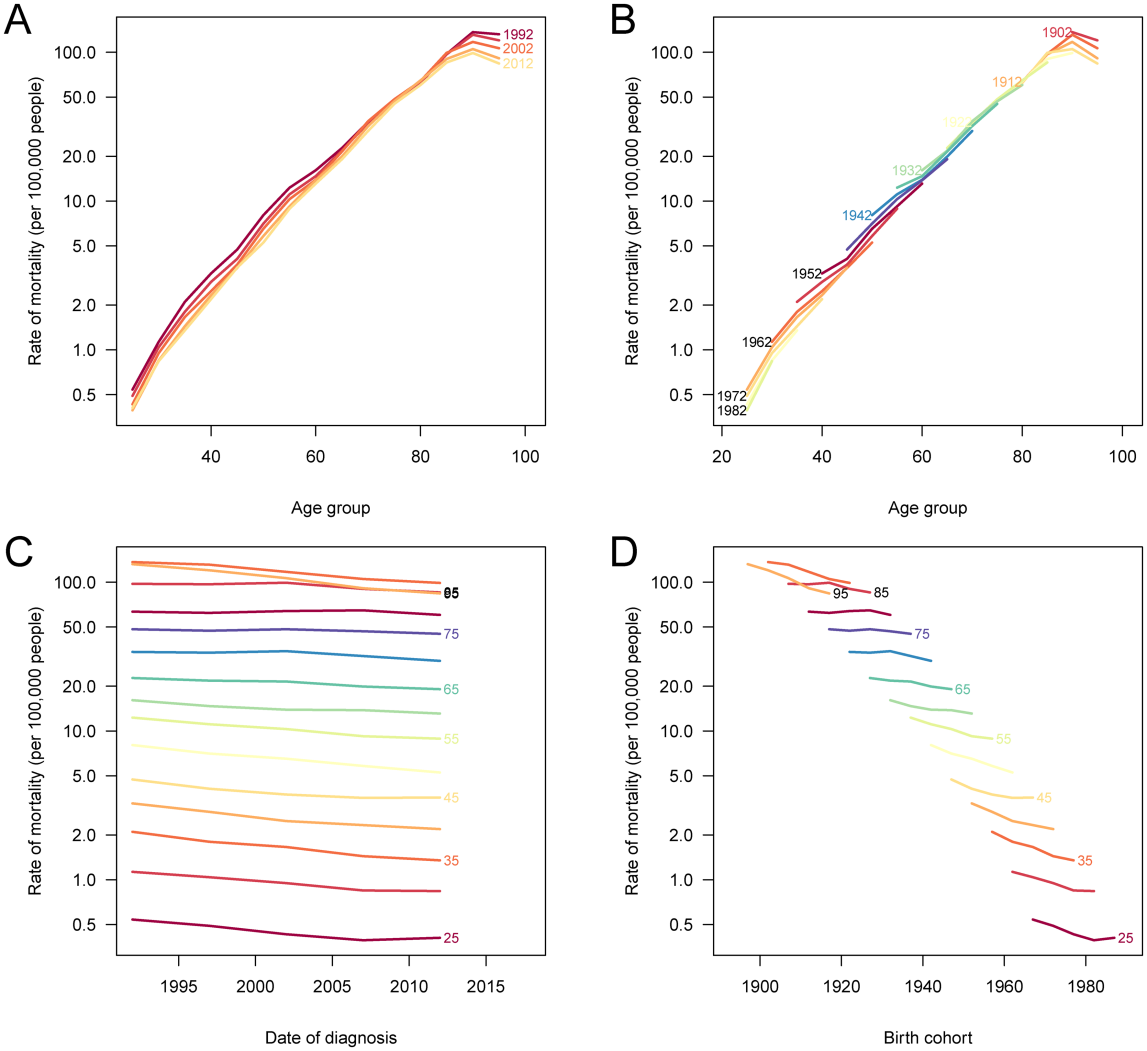
Age-period-cohort analysis of CRC mortality attributable to dietary risks in China. **(A)** The age-specific mortality rates of colon and rectum cancer attributable to dietary risks according to time periods; each line connects the age-specific mortality rate for a 5-year period. **(B)** The age-specific mortality rates of colon and rectum cancer according to birth cohort; each line connects the age-specific mortality rate for a 5-year cohort. **(C)** The period-specific mortality rates according to age groups; each line connects the birth cohort-specific mortality rate for a 5-year age group. **(D)** The birth cohort-specific mortality rates according to age groups; each line connects the birth cohort-specific mortality rate for a 5-year age group. Abbreviations: CRC, colon and rectum cancer.

Age-specific mortality and DALY rates increased substantially with advancing age, particularly among individuals aged 60 years and older, indicating that aging remains a dominant risk factor in CRC burden. Across different time periods, the mortality and DALY rates remained relatively stable or showed mild declines in recent years, suggesting gradual improvements in dietary patterns, early detection, and healthcare interventions. A pronounced cohort effect was observed, with earlier birth cohorts, especially those born before 1950, experiencing markedly higher mortality and DALY rates. In contrast, more recent cohorts exhibited a steady decline in these indicators, pointing to generational improvements in nutrition, health literacy, and access to preventive services. Additionally, age-specific rates across successive birth cohorts revealed that individuals of the same age had lower mortality and DALY rates in younger generations, further confirming the downward trend in burden over time. These findings emphasize the strong influence of age, while also reflecting positive effects of public health and dietary interventions across cohorts (Figure 6 and Supplementary Figure S3).

### Decomposition of changes in CRC burden attributable to dietary risks in China

**Figure 7:**
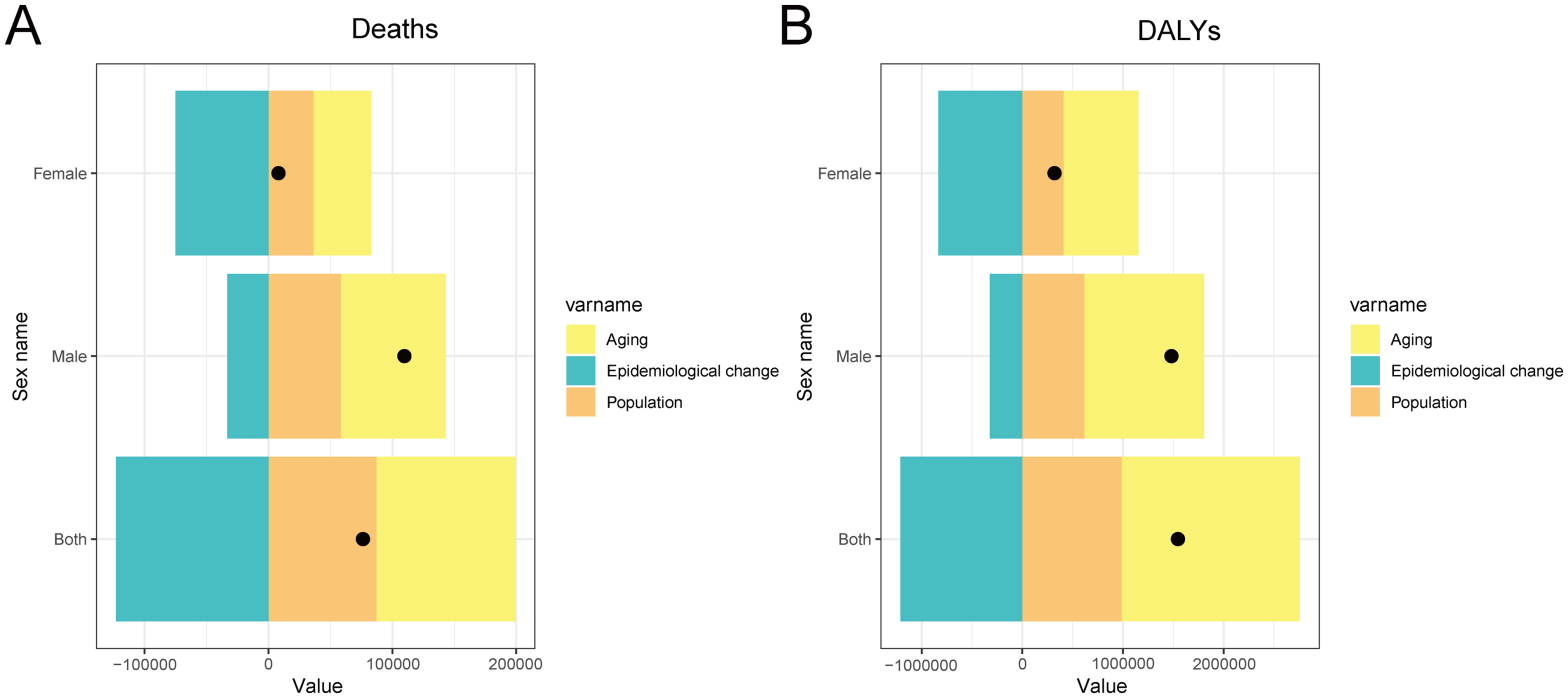
Decomposition analysis of changes in deaths and DALYs for CRC attributable to dietary risks in China from 1990 to 2021, by sex. **(A)** Contributions of population growth, population aging, and epidemiological changes to the absolute change in the number of deaths. **(B)** Contributions of the same three factors to the change in DALYs. DALY, disability-adjusted life year; CRC, colon and rectum cancer.

Figure 7 presents the decomposition analysis of the absolute changes in deaths and DALYs for CRC attributable to dietary risks in China between 1990 and 2021, stratified by sex. The findings reveal that population aging was the primary contributor to the increase in both deaths and DALYs, especially among males, underscoring the growing burden of CRC in an aging society. Population growth also played a substantial role in elevating the total burden across both sexes. In contrast, epidemiological changes reflecting shifts in dietary exposures, advances in disease detection, and improved treatment were linked to reductions in deaths and DALYs, especially among females, suggesting the effectiveness of public health initiatives and growing awareness of nutritional health. The combined effects of demographic and epidemiological transitions underscore the importance of adjusting cancer prevention strategies to address the dual pressures of an aging population and modifiable dietary risk factors. These results emphasize the urgent need for targeted dietary interventions and cancer control policies, especially for aging male populations (Figure 7).

## Discussion

This study provides a comprehensive assessment of CRC burden attributable to dietary risks in China from 1990 to 2021 using GBD data. The absolute numbers of CRC-related deaths, DALYs, YLDs, and YLLs increased markedly, especially among individuals aged 45 years and older. However, age-standardized mortality and DALY rates declined in recent years, indicating progress in screening and treatment. Processed and red meat intake, along with inadequate consumption of whole grains, milk, fiber, and calcium, were the leading dietary contributors. Age-period-cohort modeling showed elevated mortality and DALY rates among those born before 1950, while younger cohorts benefited from improved health infrastructure. Decomposition analysis identified population aging as the main driver of increased burden, partially offset by epidemiological improvements. Stratified results highlighted urban males over 40 as a particularly high-risk group. These findings underscore the evolving dietary landscape’s role in CRC burden and call for targeted, data-driven public health strategies and dietary policy interventions.

A growing body of evidence links diet to colorectal carcinogenesis through several complementary mechanisms. High intake of processed and red meat is associated with elevated CRC risk due to carcinogens generated during high-temperature cooking and curing, including heterocyclic amines, polycyclic aromatic hydrocarbons, and N-nitroso compounds (25–27). Heme iron further promotes oxidative stress and mucosal inflammation, facilitating DNA damage and epithelial proliferation (28), while N-glycolylneuraminic acid (Neu5Gc) may sustain chronic immune activation in the colonic mucosa (25, 29). Conversely, insufficient intake of dietary fiber, whole grains, calcium, and milk removes important protective pathways (30–33). Fiber increases stool bulk and shortens transit time, diluting luminal carcinogens (34); its fermentation yields butyrate, which supports epithelial integrity and exerts anti-inflammatory and anti-proliferative effects (35, 36).

Building on these pathways, the diet–microbiome–inflammation–carcinogenesis axis provides an integrative framework. Diets high in red and processed meats and low in plant fibers can reshape the gut microbiota, reduce diversity, and favor pro-inflammatory taxa, thereby increasing intestinal permeability and endotoxemia and sustaining a tumor-promoting inflammatory milieu (15, 37–40). Calcium can bind secondary bile acids and free fatty acids, lowering mucosal toxicity (41), and dairy-derived constituents such as vitamin D and lactoferrin may confer additional antitumor activity (42, 43). Our observation that low milk and low calcium contribute substantially to CRC burden is consistent with these protective mechanisms. To strengthen causal inference, future studies should integrate longitudinal diet assessment with microbiome and inflammatory biomarkers and incorporate validation in gnotobiotic or germ-free models. Collectively, these data underscore the value of dietary patterns rich in protective components and low in pro-carcinogenic exposures for CRC prevention.

The disparities in CRC burden attributable to dietary risks observed across gender, dietary drivers, and birth cohorts are multifactorial. Notably, men consistently exhibited higher age-standardized mortality and DALY rates than women, which may reflect a combination of behavioral, biological, and social factors. Men generally consume more red and processed meat and less fiber, dairy, and calcium-rich foods, resulting in greater cumulative exposure to adverse dietary risks. Culturally, men in China are also more likely to engage in behaviors such as heavy alcohol consumption and smoking, which may compound the carcinogenic effects of poor dietary habits (44–47). Additionally, men often demonstrate lower health-seeking behavior, with reduced participation in preventive screening and dietary counseling programs, potentially leading to delayed diagnosis and worse outcomes (48). Conversely, women’s higher nutritional awareness and healthcare engagement may facilitate earlier detection and dietary modification; estrogenic signaling may also confer anti-inflammatory, tumor-suppressive effects (49).

With regard to specific dietary drivers, diets high in processed and red meat were more strongly associated with CRC-related deaths and DALYs, particularly among older adults. This pattern is consistent with cumulative exposure to dietary carcinogens and age-related decline in DNA repair capacity (50). The cohort analysis indicated that individuals born before 1950 experienced significantly higher CRC mortality and DALY rates attributable to dietary risks. These patterns likely stem from historical dietary habits marked by inadequate consumption of fiber and dairy products, coupled with a heavier reliance on preserved and red meats. Such trajectories were shaped by wartime scarcity, rapid post-war industrialization, and limited nutrition literacy (51); more recent cohorts appear to benefit from dietary diversification, health campaigns, and improved literacy (52). Persisting high burden in select groups highlights CRC’s long latency and the need for sustained, population-specific prevention.

The observed trends in CRC burden attributable to dietary risks carry critical implications for both clinical practice and public health policy. Clinically, the findings underscore the importance of incorporating brief, structured dietary risk assessments into routine CRC prevention and management strategies. Primary care and oncology providers should be encouraged to evaluate patients’ dietary patterns and offer evidence-based nutritional counseling, particularly for individuals at high risk due to family history or comorbidities (11). Moreover, given the strong association between processed/red meat consumption and CRC outcomes, dietary modification should be embedded in survivorship care plans. In light of these findings, current CRC screening guidelines may warrant refinement (53). Individuals with sustained high-risk dietary patterns (high processed/red meat, low fiber/dairy/whole grains) may merit earlier initiation and/or more frequent screening; risk-adapted protocols that integrate diet with family history, lifestyle, and comorbidities could improve early detection and cost-effectiveness (54).

From a policy perspective, the increasing burden attributable to modifiable dietary factors signals an urgent need for national strategies aimed at promoting healthier diets. Priority options include processed-meat sodium reduction, front-of-package warning labels, and targeted subsidies for dairy and fiber-rich foods, evaluated through policy simulation for feasibility and impact (55). These may include public education campaigns, food labeling reforms, and taxation or regulation of processed meats, which have shown promise in other settings (56). Schools and workplaces should also be targeted to encourage environments conducive to healthy eating, including access to whole grains, dairy, and fiber-rich foods. In China, rapid urbanization and Westernization of diets have contributed to rising CRC incidence, particularly among younger cohorts (57, 58). Accordingly, age-tailored prevention should be prioritized (59). Regional adaptation of these policies could maximize effectiveness in resource-limited or culturally distinct areas. Additionally, disparities across gender and regions suggest the need for geographically and culturally adapted policies that account for local dietary habits and healthcare access. Ultimately, integrating nutritional interventions into national cancer control programs and non-communicable disease frameworks could offer a cost-effective means to curb the CRC burden and improve population health.

This study has several limitations that warrant consideration. First, the analysis is based on secondary estimates from the GBD 2021 study, which synthesizes data from heterogeneous sources. Although robust modeling techniques are used, potential biases from data imputation, reporting inconsistencies, and regional gaps in cancer registration may affect accuracy, especially in under-resourced areas of China. In particular, cancer registries in China exhibit greater coverage in urban regions compared to rural ones, which may lead to underestimation of CRC burden in less developed areas and obscure geographic disparities in disease patterns (60). Second, the attribution of CRC burden to dietary risks is based on comparative risk assessment models, which infer causality from observational data. While associations are biologically plausible, residual confounding from lifestyle, genetic, or socioeconomic factors remains possible. Third, population-level exposure estimates may not capture individual variation in intake, preparation, or processing. Fourth, the ecological nature of the study limits causal inference at the individual level. Fifth, while we applied age-period-cohort and decomposition analyses, results may be influenced by model assumptions and collinearity. Future research should incorporate prospective cohorts with biomarker and microbiome endpoints and conduct policy-evaluation or intervention trials to quantify the effectiveness of diet-focused prevention.

## Conclusion

This study underscores the substantial public health challenge posed by CRC attributable to dietary risks in China. The persistent burden reflects both historical dietary patterns and emerging nutritional transitions. Addressing this issue requires sustained attention to dietary quality, especially in the context of an aging population and evolving lifestyle behaviors. Strengthening dietary guidelines, implementing population-level interventions, and enhancing nutrition education are critical components of a comprehensive cancer prevention strategy. Furthermore, incorporating nutritional surveillance into routine public health monitoring may facilitate timely adjustments in policy and practice. Future research should focus on high-resolution dietary assessments, the exploration of gene-diet interactions, and the development of targeted dietary interventions for high-risk groups. In addition, translational studies that connect dietary exposures to molecular and microbiome alterations in colorectal carcinogenesis will provide important mechanistic insights. Ultimately, an integrated approach combining epidemiology, clinical research, and policy action will be essential to reduce the diet-related CRC burden.

## Data Availability

Publicly available datasets were analyzed in this study. This data can be found at: https://vizhub.healthdata.org/gbd-results/.

## Supplementary material

SUPPLEMENTARY FIGURE S1 Comparison of age-specific numbers and crude rates of **(A)** deaths, **(B)** DALYs, **(C)** YLDs, and **(D)** YLLs for CRC attributable to dietary risks in China, between 1990 and 2021. DALYs, disability-adjusted life years; YLDs, years lived with disability; YLLs, years of life lost; CRC, colon and rectum cancer.

SUPPLEMENTARY FIGURE S2 Trends in age-standardized rates of mortality, DALYs, YLDs, and YLLs for CRC attributable to dietary risks in China **(A)** and globally **(B)**, 1990–2021. DALYs, disability-adjusted life years; YLDs, years lived with disability; YLLs, years of life lost; CRC, colon and rectum cancer.

SUPPLEMENTARY FIGURE S3 Age-period-cohort analysis of DALYs for colon and rectum cancer attributable to dietary risks in China. **(A)** The age-specific DALY rates of colon and rectum cancer according to time periods; each line connects the age-specific DALY rate for a 5-year period. **(B)** The age-specific DALY rates according to birth cohort; each line connects the age-specific DALY rate for a 5-year cohort. **(C)** The period-specific DALY rates according to age groups; each line connects the birth cohort-specific DALY rate for a 5-year age group. **(D)** The birth cohort-specific DALY rates according to age groups; each line connects the birth cohort-specific DALY rate for a 5-year age group. DALY, disability-adjusted life year; CRC, colon and rectum cancer.
