# Supplementary figures and images for: Burden of colorectal cancer attributable to dietary risks in China from 1990 to 2021: findings from the Global Burden of Disease Study 2021
Source: Front Nutr. 2026 Jan 6;12:1673267. doi: 10.3389/fnut.2025.1673267 (PMC12815792; doi:10.3389/fnut.2025.1673267)

A

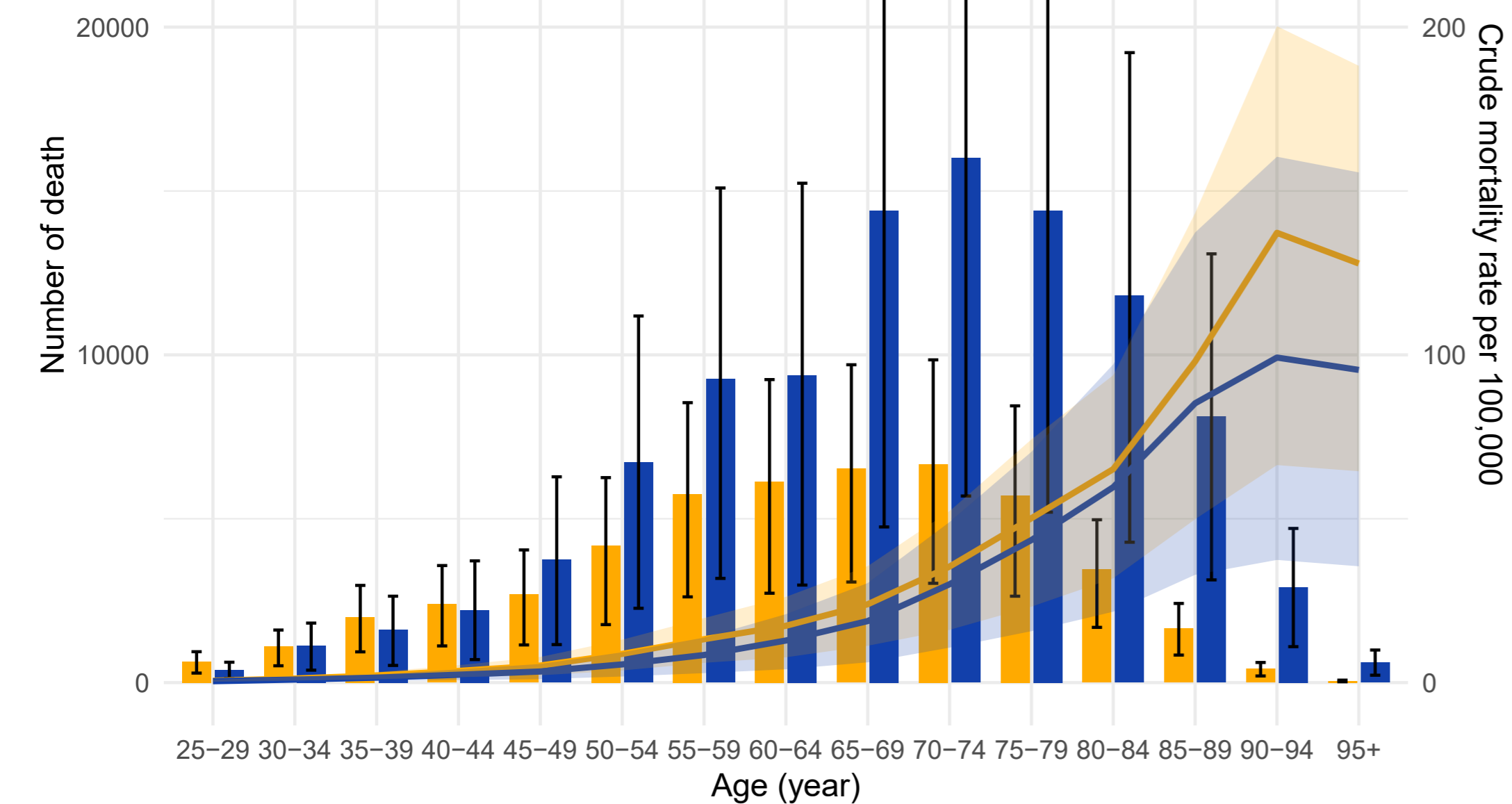

B

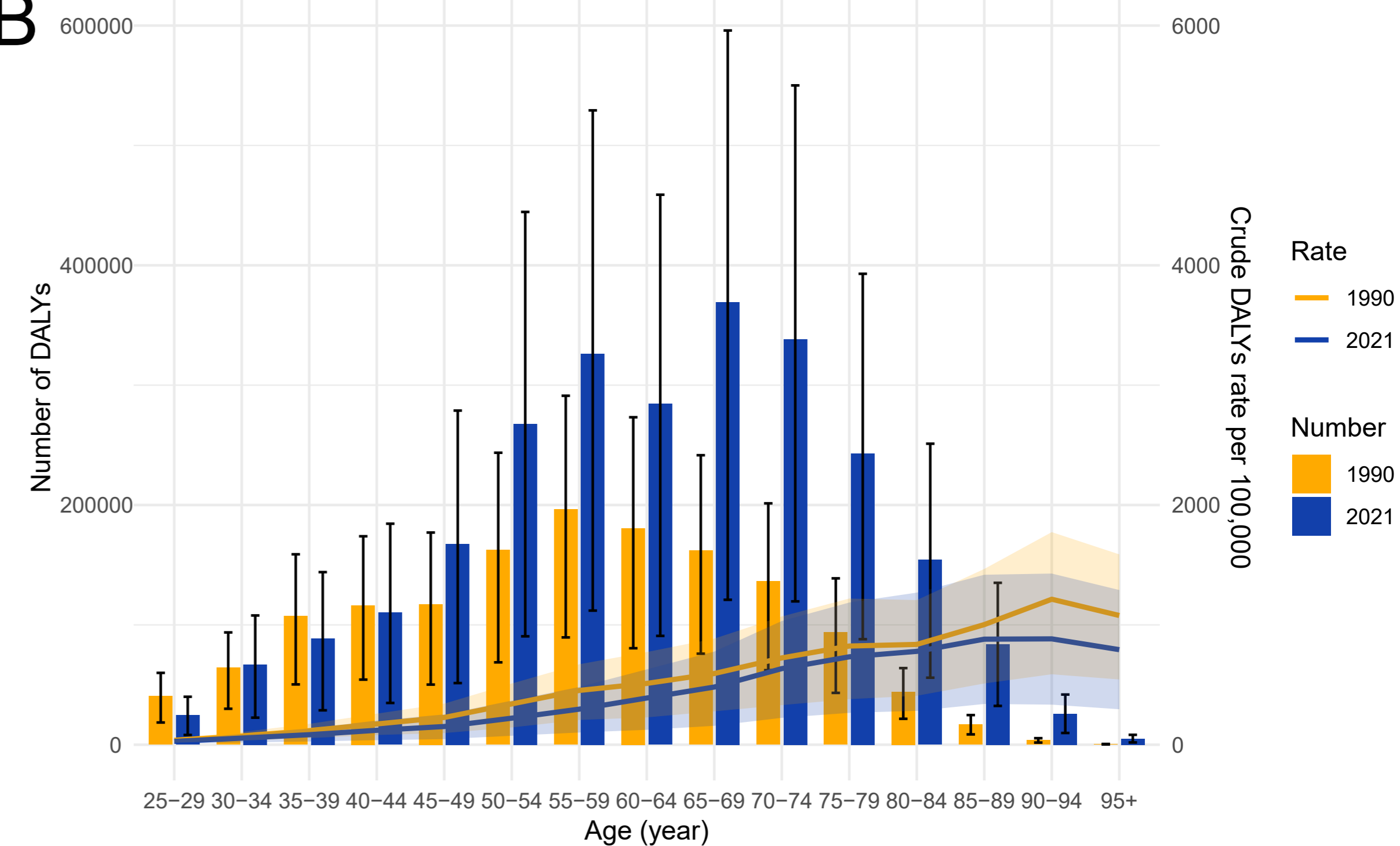

C

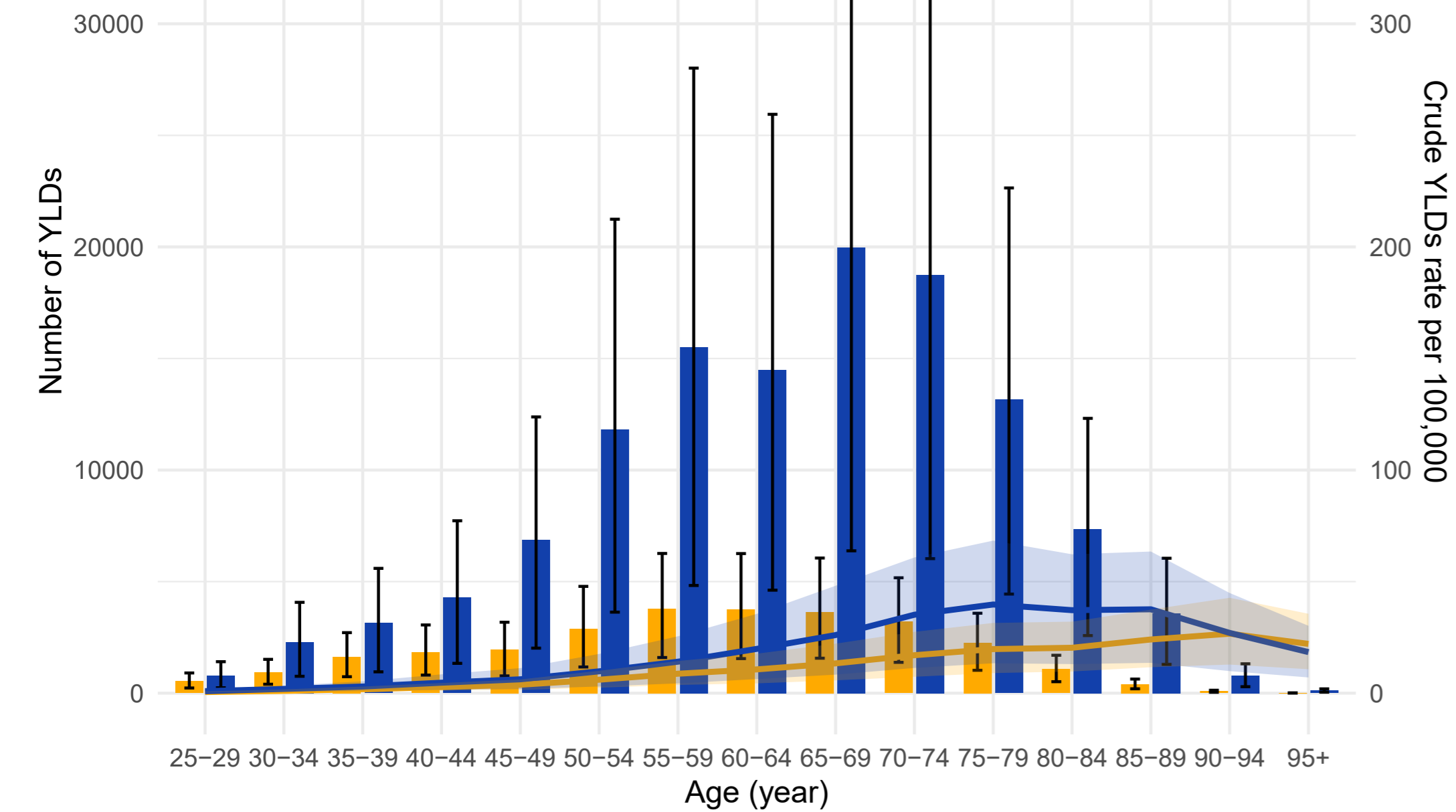

D

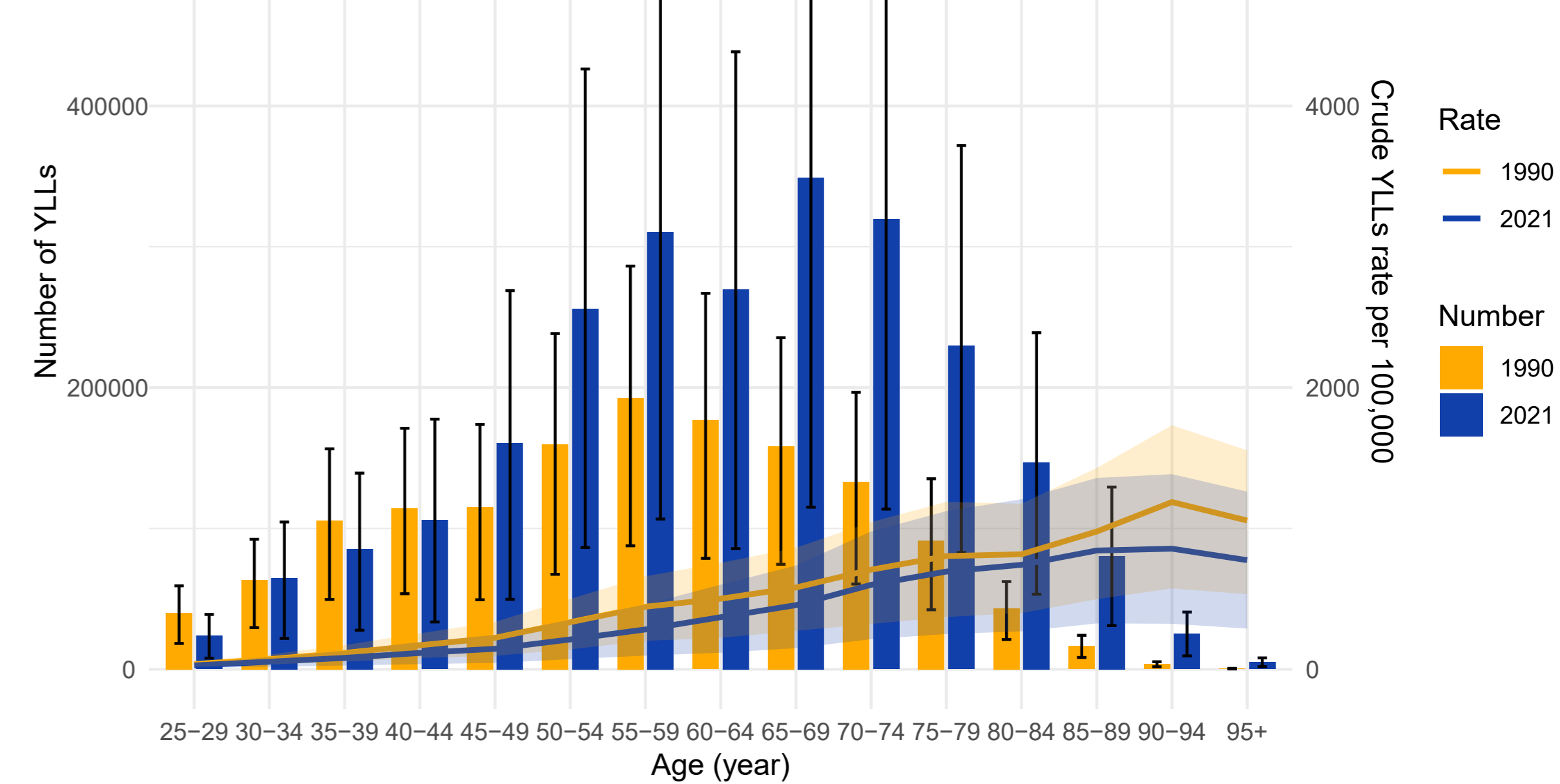

Supplement: SUPPLEMENTARY FIGURE S1 — Comparison of age-specific numbers and crude rates of (A) deaths, (B) DALYs, (C) YLDs, and (D) YLLs for CRC attributable to dietary risks in China, between 1990 and 2021. DALYs, disability-adjusted life years; YLDs, years lived with disability; YLLs, years of life lost; CRC, colon and rectum cancer. [file Data_Sheet_1.PDF]

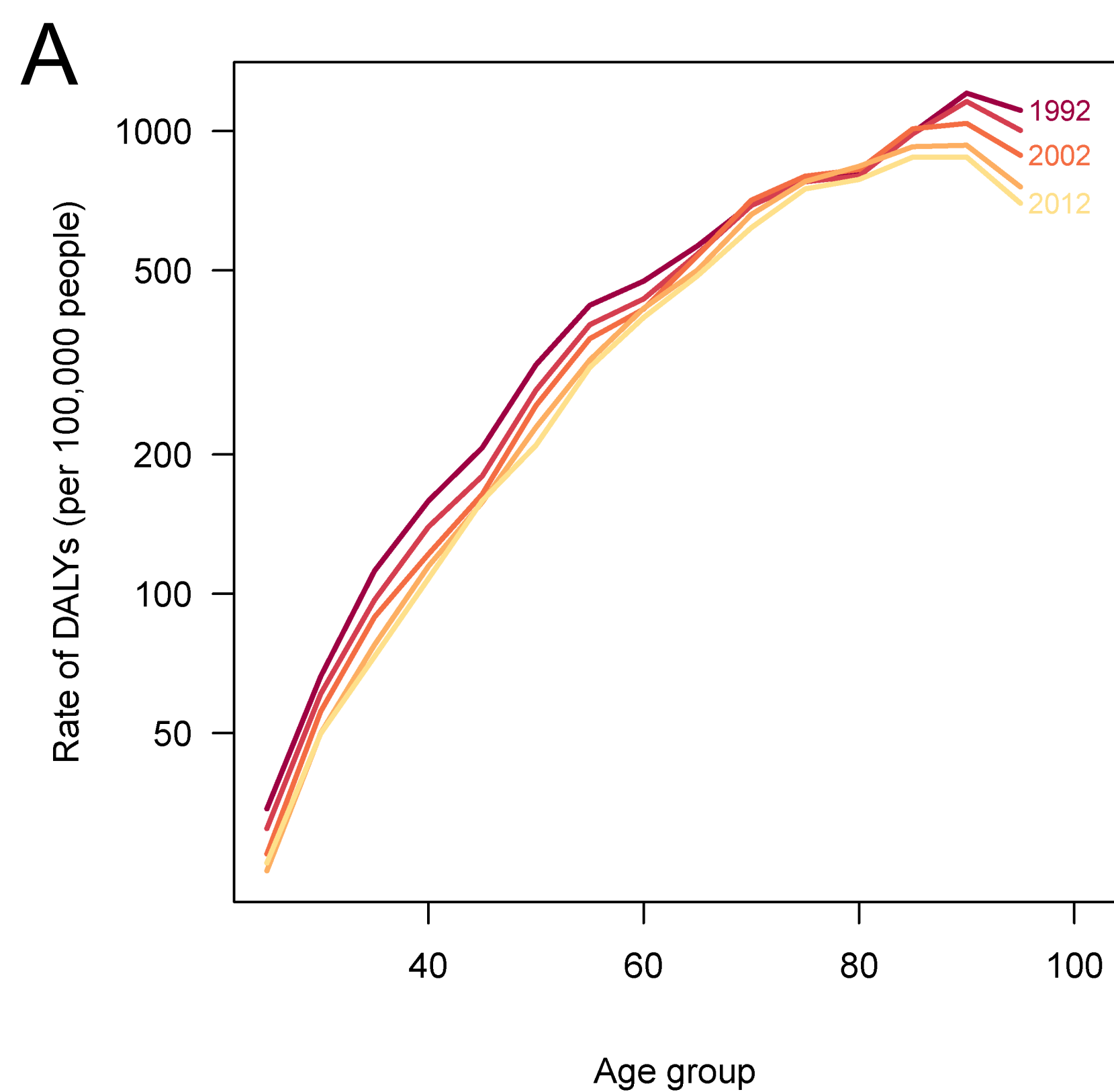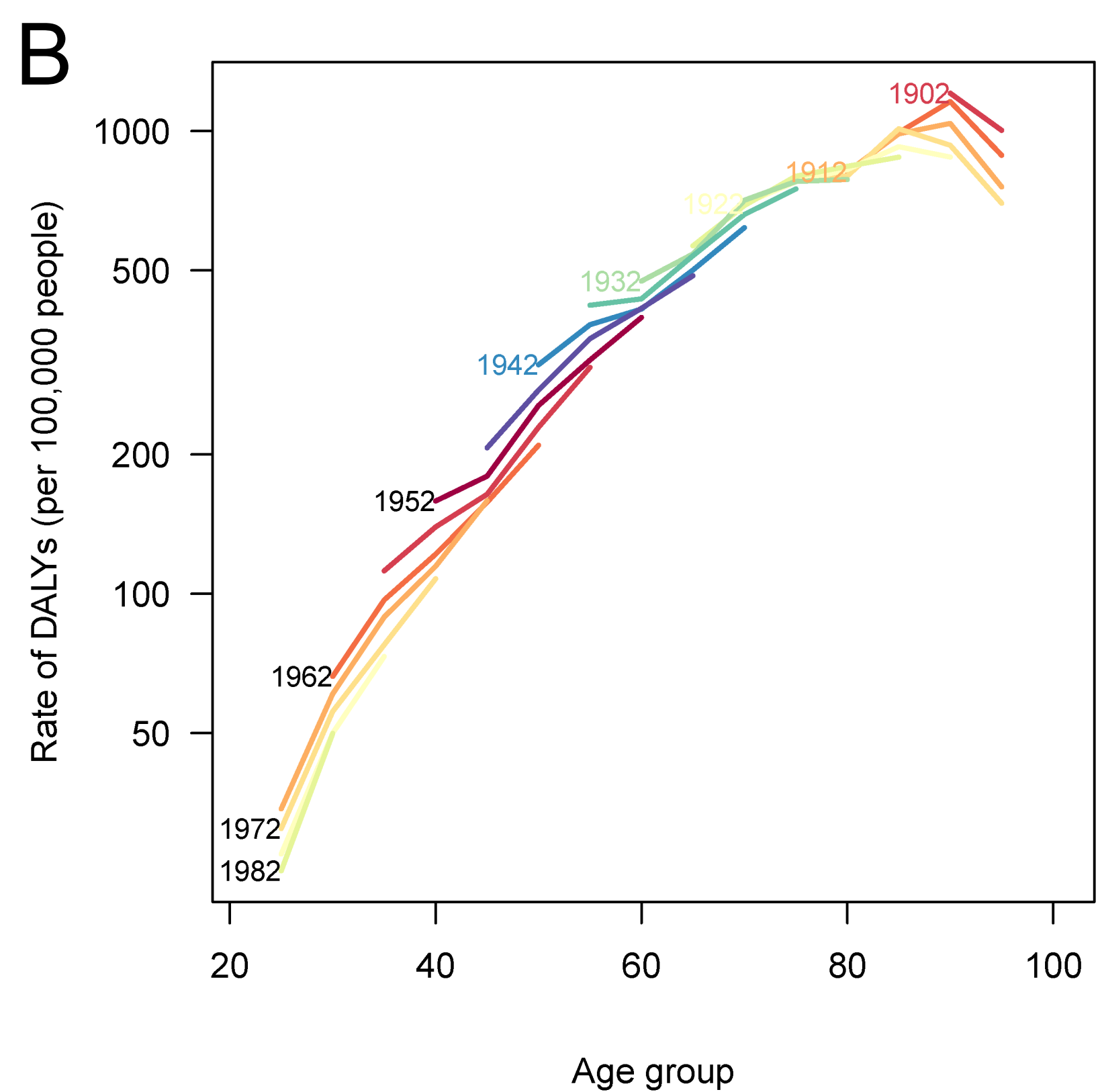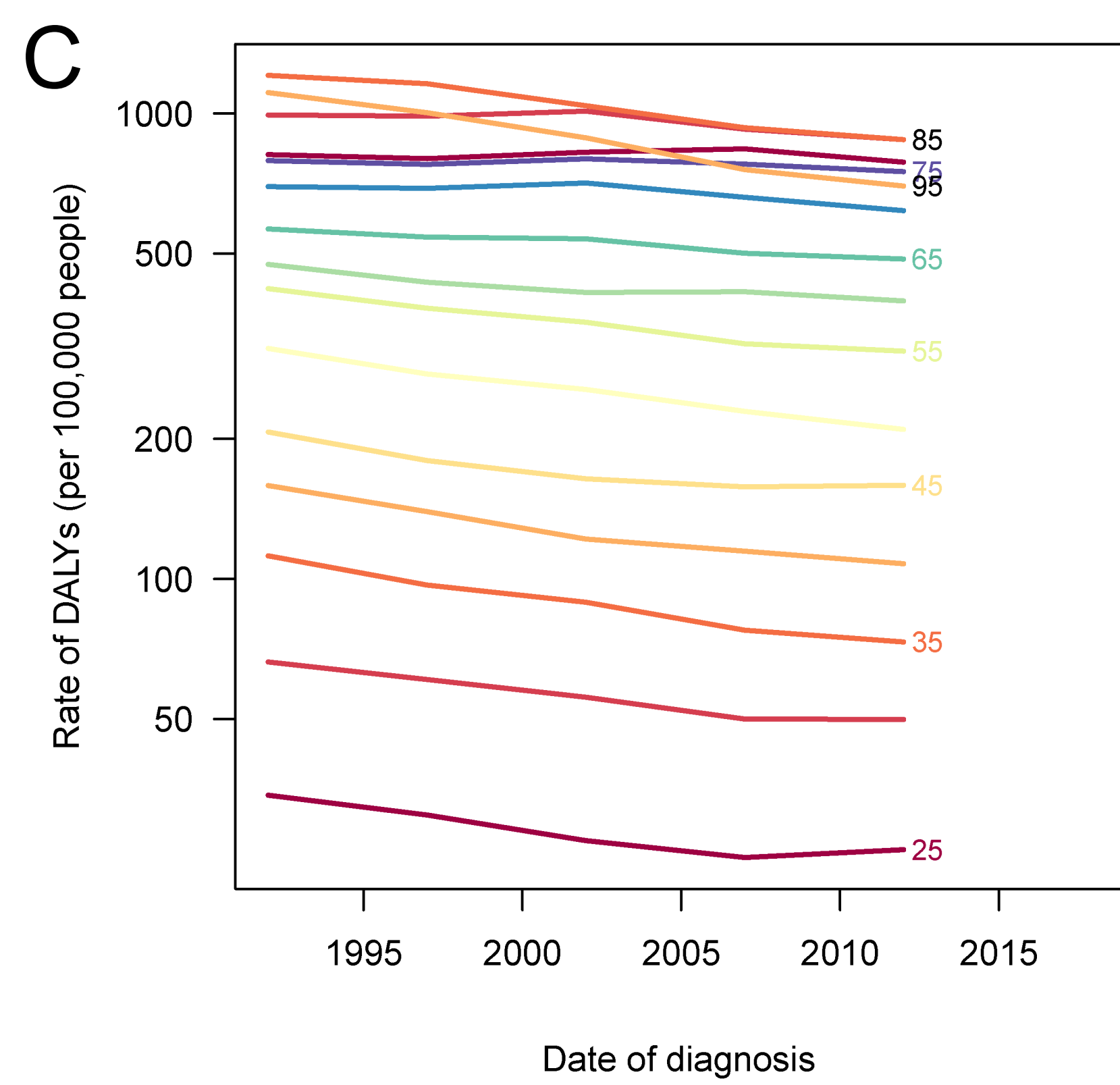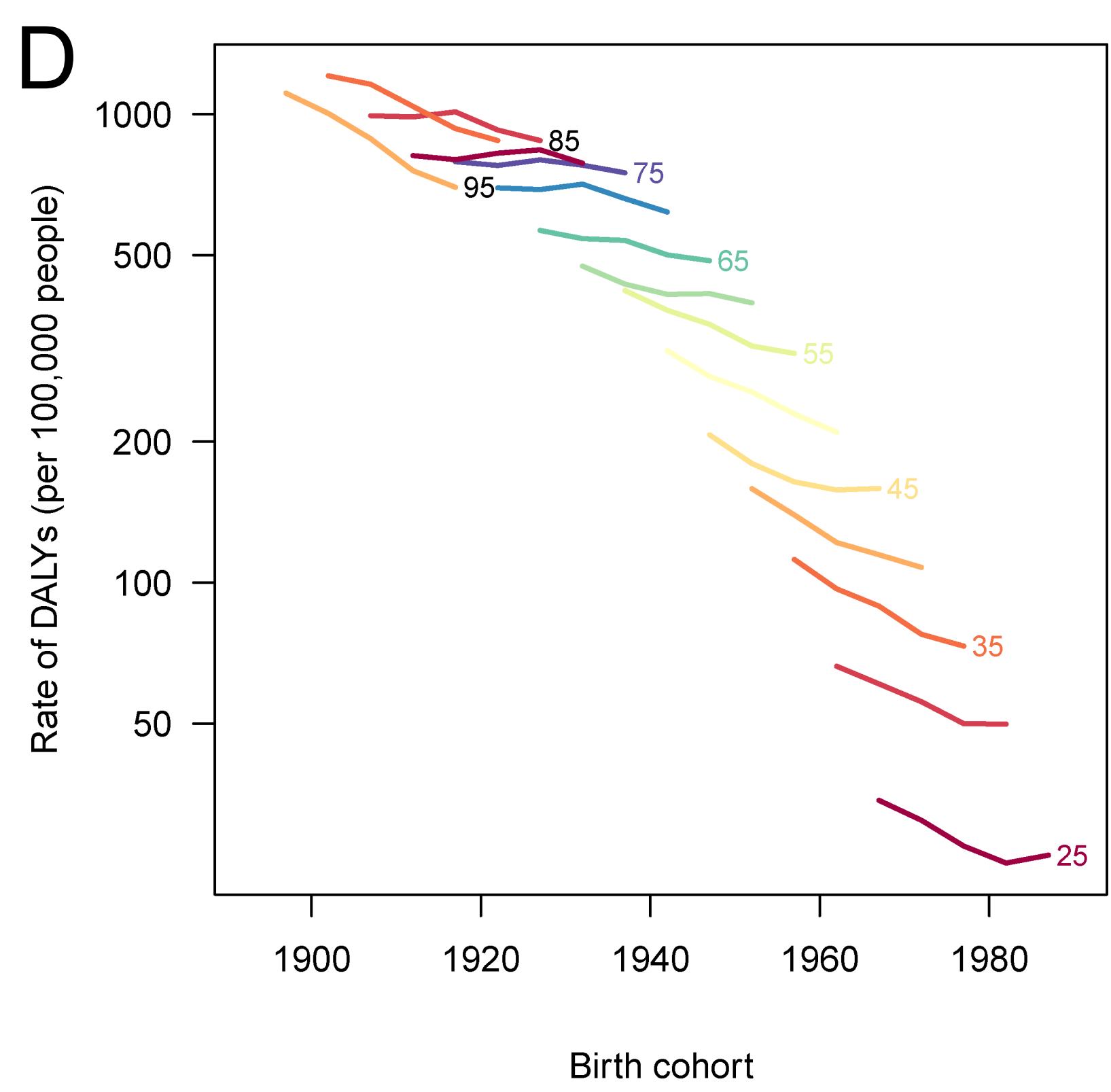

Supplement: SUPPLEMENTARY FIGURE S3 — Age-period-cohort analysis of DALYs for colon and rectum cancer attributable to dietary risks in China. (A) The age-specific DALY rates of colon and rectum cancer according to time periods; each line connects the age-specific DALY rate for a 5-year period. (B) The age-specific DALY rates according to birth cohort; each line connects the age-specific DALY rate for a 5-year cohort. (C) The period-specific DALY rates according to age groups; each line connects the birth cohort-specific DALY rate for a 5-year age group. (D) The birth cohort-specific DALY rates according to age groups; each line connects the birth cohort-specific DALY rate for a 5-year age group. DALY, disability-adjusted life year; CRC, colon and rectum cancer. [file Data_Sheet_3.PDF]
