# Supplementary material for: Burden of colorectal cancer attributable to dietary risks in China from 1990 to 2021: findings from the Global Burden of Disease Study 2021
Source: Front Nutr. 2026 Jan 6;12:1673267. doi: 10.3389/fnut.2025.1673267 (PMC12815792; doi:10.3389/fnut.2025.1673267)
Supplement: SUPPLEMENTARY FIGURE S2 — Trends in age-standardized rates of mortality, DALYs, YLDs, and YLLs for CRC attributable to dietary risks in China (A) and globally (B), 1990–2021. DALYs, disability-adjusted life years; YLDs, years lived with disability; YLLs, years of life lost; CRC, colon and rectum cancer. [file Data_Sheet_2.PDF]

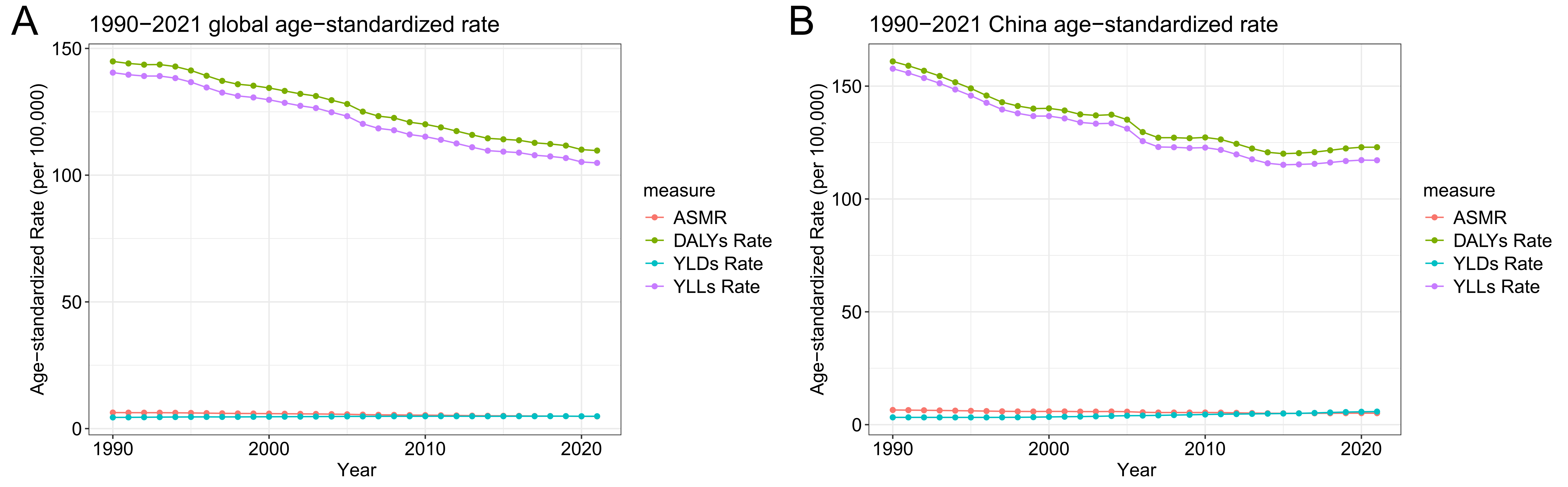
A
1990−2021 global age−standardized rate
Age−standardized Rate (per 100,000)
150
100
50
0
1990
2000
2010
2020
Year
measure
ASMR
DALYs Rate
YLDs Rate
YLLs Rate
B
1990−2021 China age−standardized rate
Age−standardized Rate (per 100,000)
150
100
50
0
1990
2000
2010
2020
Year
measure
ASMR
DALYs Rate
YLDs Rate
YLLs Rate
