# Supplementary material for: Burden of colorectal cancer attributable to dietary risks in China from 1990 to 2021: findings from the Global Burden of Disease Study 2021
Source: Front Nutr. 2026 Jan 6;12:1673267. doi: 10.3389/fnut.2025.1673267 (PMC12815792; doi:10.3389/fnut.2025.1673267)
Supplement: Supplementary file 4 [file Table_1.DOCX]

Table S1. YLDs and YLLs for CRC attributable to dietary risks in China, 2021, with trends in ASRs per 100, 000 population, 1990 - 2021

|  | YLDs | | | YLLs | | |
| --- | --- | --- | --- | --- | --- | --- |
| Dietary risk | No, in thousands | Age-standardized rate per 100, 000 | Percentage change from 1990 to 2021 | No, in thousands | Age-standardized rate per 100, 000 | Percentage change from 1990 to 2021 |
| Diet high in processed meat | 8.6 (-1.8, 19.7) | 0.4 (-0.1, 0.9) | 243.9 (165, 350.3) | 169.6 (-35.1, 387.6) | 8.2 (-1.7, 18.5) | 42.9 (9.3, 90.2) |
| Diet high in red meat | 52.6 (0, 116.4) | 2.5 (0, 5.5) | 114 (65.2, 1725.2) | 1039.2 (-0.5, 2198.1) | 50 (0, 105.7) | -11.3 (-32.7, 669.8) |
| Diet low in milk | 60.8 (15.3, 111.2) | 2.9 (0.7, 5.2) | 93.1 (50.9, 150.4) | 1192.9 (323, 2035.9) | 57.4 (15.5, 98) | -19.9 (-38.6, 4) |
| Diet low in calcium | 23.9 (15, 36.4) | 1.1 (0.7, 1.7) | -7.7 (-29.5, 23) | 476.6 (338.8, 650.5) | 23.1 (16.3, 31.5) | -61.8 (-71.1, -49.5) |
| Diet low in fiber | 2.2 (0.9, 4.2) | 0.1 (0, 0.2) | -23 (-49.4, 9.7) | 45.9 (18, 81) | 2.3 (0.9, 4) | -68.1 (-79.1, -53.9) |
| Diet low in whole grains | 59.8 (22.1, 101.5) | 2.8 (1, 4.8) | 101.1 (56.5, 159.9) | 1182.1 (478.4, 1889.5) | 56.9 (23, 90.8) | -16.5 (-37.2, 7.6) |

Values in parentheses indicate 95% UIs estimated from Monte Carlo simulations using GBD posterior draws. ASRs are per 100,000 population and age-standardized to the GBD standard population. Abbreviations: YLDs, years lived with disability; YLLs, years of life lost; ASRs, age-standardized rates; UI, uncertainty interval.
