# Supplementary material for: Burden of colorectal cancer attributable to dietary risks in China from 1990 to 2021: findings from the Global Burden of Disease Study 2021
Source: Front Nutr. 2026 Jan 6;12:1673267. doi: 10.3389/fnut.2025.1673267 (PMC12815792; doi:10.3389/fnut.2025.1673267)
Supplement: Supplementary file 5 [file Table_2.DOCX]

Table S2. Trends in age-standardized mortality, DALY, YLD, and YLL rates (per 100,000 persons) among both sexes, males, and females from 1990 to 2021 for CRC attributable to dietary risks in China.

|  | Age-standardized mortality rate | | | Age-standardized DALY rate | | | Age-standardized YLD rate | | | Age-standardized YLL rate | | | |
| --- | --- | --- | --- | --- | --- | --- | --- | --- | --- | --- | --- | --- | --- |
| Gender | Period | APC (95% CI) | AAPC (95% CI) | Period | APC (95% CI) | AAPC (95% CI) | Period | APC (95% CI) | AAPC (95% CI) | Period | APC (95% CI) | AAPC (95% CI) |  |
| Both | 1990-1998 | -1.37 (-1.53 - -1.24) ^***^ | -0.81 (-0.84 - -0.78) ^***^ | 1990-1998 | -1.71 (-1.86 - -1.60) ^***^ | -0.88 (-0.91 - -0.85) ^***^ | 1990-1998 | 0.03 (-0.09 - 0.14) | 1.92 (1.87 - 1.96) ^***^ | 1990-1998 | -1.75 (-1.89 - -1.64) ^***^ | -0.97 (-1.00 - -0.94) ^***^ |  |
|  | 1998-2004 | -0.09 (-0.29 - 0.23) |  | 1998-2004 | -0.48 (-0.64 - -0.18) ^*^ |  | 1998-2011 | 2.80 (1.04 - 2.87) ^*^ |  | 1998-2004 | -0.56 (-0.73 - -0.29) ^**^ |  |  |
|  | 2004-2007 | -2.59 (-2.95 - -1.97) ^***^ |  | 2004-2007 | -2.50 (-2.82 - -1.99) ^***^ |  | 2011-2016 | 1.62 (0.99 - 2.86) ^***^ |  | 2004-2007 | -2.65 (-2.96 - -2.16) ^***^ |  |  |
|  | 2007-2011 | -0.07 (-0.34 - 0.52) |  | 2007-2010 | 0.08 (-0.37 - 0.41) |  | 2016-2019 | 3.74 (1.56 - 4.19) ^***^ |  | 2007-2010 | -0.02 (-0.46 - 0.29) |  |  |
|  | 2011-2014 | -2.16 (-2.50 - -1.52) ^***^ |  | 2010-2015 | -1.24 (-1.76 - -1.02) ^***^ |  | 2019-2021 | 1.81 (1.04 - 3.04) ^***^ |  | 2010-2015 | -1.35 (-1.84 - -1.15) ^***^ |  |  |
|  | 2014-2021 | 0.17 (-0.00 - 0.39) |  | 2015-2021 | 0.50 (0.28 - 0.79) ^***^ |  |  |  |  | 2015-2021 | 0.39 (0.18 - 0.66) ^**^ |  |  |
| Female | 1990-1998 | -1.67 (-2.18 - -1.50) ^***^ | -1.36 (-1.40 - -1.32) ^***^ | 1990-2004 | -1.86 (-1.93 - -1.79) ^***^ | -1.53 (-1.56 - -1.50) ^***^ | 1990-1998 | -0.10 (-0.45 - 0.14) | 1.36 (1.29 - 1.41) ^***^ | 1990-2004 | -1.92 (-1.99 - -1.85) ^***^ | -1.63 (-1.66 - -1.60) ^***^ |  |
|  | 1998-2004 | -1.15 (-1.40 - -0.60) ^***^ |  | 2004-2007 | -3.13 (-3.45 - -2.45) ^***^ |  | 1998-2009 | 1.82 (-0.10 - 2.02) |  | 2004-2007 | -3.33 (-3.66 - -2.69) ^***^ |  |  |
|  | 2004-2007 | -3.20 (-3.64 - -2.43) ^***^ |  | 2007-2011 | -1.69 (-2.16 - -1.14) ^***^ |  | 2009-2015 | 0.79 (0.24 - 2.09) ^*^ |  | 2007-2011 | -1.79 (-2.24 - -1.23) ^***^ |  |  |
|  | 2007-2011 | -1.49 (-1.88 - -0.84) ^***^ |  | 2011-2014 | -2.92 (-3.29 - -2.06) ^*^ |  | 2015-2019 | 3.62 (0.54 - 4.48) ^**^ |  | 2011-2014 | -3.04 (-3.41 - -2.29) ^*^ |  |  |
|  | 2011-2014 | -3.23 (-3.64 - -2.37) ^*^ |  | 2014-2021 | 0.54 (0.34 - 0.79) ^**^ |  | 2019-2021 | 1.89 (0.81 - 3.25) ^***^ |  | 2014-2021 | 0.43 (0.23 - 0.67) ^**^ |  |  |
|  | 2014-2021 | 0.52 (0.28 - 0.78) ^*^ |  |  |  |  |  |  |  |  |  |  |  |
| Male | 1990-1998 | -1.12 (-1.31 - -0.97) ^**^ | -0.42 (-0.46 - -0.38) ^***^ | 1990-1998 | -1.50 (-1.62 - -1.39) ^***^ | -0.43 (-0.46 - -0.40) ^***^ | 1990-1998 | 0.11 (-0.07 - 0.29) | 2.28 (2.23 - 2.33) ^***^ | 1990-1998 | -1.53 (-1.67 - -1.41) ^***^ | -0.52 (-0.55 - -0.49) ^***^ |  |
|  | 1998-2004 | 0.84 (0.61 - 1.21) ^**^ |  | 1998-2004 | 0.48 (0.31 - 0.71) ^***^ |  | 1998-2011 | 3.52 (3.40 - 3.64) ^***^ |  | 1998-2004 | 0.40 (0.22 - 0.66) ^***^ |  |  |
|  | 2004-2007 | -2.14 (-2.53 - -1.39) ^**^ |  | 2004-2007 | -1.88 (-2.20 - -1.31) ^***^ |  | 2011-2021 | 2.45 (2.21 - 2.64) ^***^ |  | 2004-2007 | -2.03 (-2.37 - -1.44) ^***^ |  |  |
|  | 2007-2011 | 0.67 (0.32 - 1.34) ^**^ |  | 2007-2011 | 0.78 (0.45 - 1.31) ^***^ |  |  |  |  | 2007-2011 | 0.67 (0.34 - 1.22) ^***^ |  |  |
|  | 2011-2014 | -1.51 (-1.92 - -0.78) ^**^ |  | 2011-2015 | -0.90 (-1.42 - -0.50) ^***^ |  |  |  |  | 2011-2015 | -1.01 (-1.54 - -0.60) ^***^ |  |  |
|  | 2014-2021 | -0.09 (-0.28 - 0.34) |  | 2015-2021 | 0.34 (0.12 - 0.73) ^**^ |  |  |  |  | 2015-2021 | 0.24 (0.01 - 0.66) ^*^ |  |  |

Abbreviations: CRC, colon and rectum cancer; DALYs, disability-adjusted life-years; YLDs, years lived with disability; YLLs, years of life lost; AAPC, average annual percent change presented for full period; APC, annual percent change; CI, confidence interval. Significance codes: *p* <0.05 (*), *p* <0.01 (**), *p* <0.001 (***). Symbols shown for APC within periods and AAPC overall; exact *p* values available upon request.
